# Genome-wide identification and evolution of HECT genes in wheat

**DOI:** 10.7717/peerj.10457

**Published:** 2020-12-02

**Authors:** Xianwen Meng, Ting Yang, Jing Liu, Mingde Zhao, Jiuli Wang

**Affiliations:** The College of Ecological Environmental and Resources, Qinghai Provincial Key Laboratory of High Value Utilization of Characteristic Economic Plants, Qinghai Nationalities University, Xining, China

**Keywords:** Wheat, HECT genes, Evolution, Segmental duplication, Expression

## Abstract

**Background:**

As an important class of E3 ubiquitin ligases in the ubiquitin proteasome pathway, proteins containing homologous E6-AP carboxyl terminus (HECT) domains are crucial for growth, development, metabolism, and abiotic and biotic stress responses in plants. However, little is known about *HECT* genes in wheat (*Triticum aestivum* L.), one of the most important global crops.

**Methods:**

Using a genome-wide analysis of high-quality wheat genome sequences, we identified 25 *HECT* genes classified into six groups based on the phylogenetic relationship among wheat, rice, and *Arabidopsis thaliana*.

**Results:**

The predicted *HECT* genes were distributed evenly in 17 of 21 chromosomes of the three wheat subgenomes. Twenty-one of these genes were hypothesized to be segmental duplication genes, indicating that segmental duplication was significantly associated with the expansion of the wheat* HECT* gene family. The Ka/Ks ratios of the segmental duplication of these genes were less than 1, suggesting purifying selection within the gene family. The expression profile analysis revealed that the 25 wheat *HECT* genes were differentially expressed in 15 tissues, and genes in Group II, IV, and VI (*UPL8*, *UPL6*, *UPL3*) were highly expressed in roots, stems, and spikes. This study contributes to further the functional analysis of the *HECT* gene family in wheat.

## Introduction

Ubiquitination is a post-translational modification that involves the covalent attachment of ubiquitin to a protein substrate. Ubiquitination is important for cellular homeostatic maintenance and plays essential roles in plant growth, development, and the regulation of abiotic and biotic stresses (Downes et al., 2003; El Refy et al., 2003; Liu & Stone, 2011; Miao & Zentgraf, 2010; Moon, Parry & Estelle, 2004; Rotin & Kumar, 2009; Stone, 2014; Wang & Deng, 2011). At the end of a three-enzyme cascade (E1 ubiquitin activating enzyme; E2 ubiquitin conjugating enzyme; E3 ubiquitin ligase), E3 recruits substrates and promotes or directly catalyzes ubiquitin transfer onto its targets (Huibregtse et al., 1995; Scheffner, Nuber & Huibregtse, 1995; Wang et al., 2006; Wang & Pickart, 2005). E3 generally determines the specificity of the ubiquitination reaction with different substrate recognition domains (Huibregtse et al., 1995; Kim et al., 2007; Scheffner, Nuber & Huibregtse, 1995). According to structural similarities and ubiquitination domains, plant E3 proteins can be classified as three main types (Chen & Hellmann, 2013; Craig et al., 2009; Duplan & Rivas, 2014; Guzman, 2014; Huibregtse et al., 1995; Mach, 2008; Maspero et al., 2013; Qin et al., 2008; Scheffner, Nuber & Huibregtse, 1995; Schwechheimer & Calderon Villalobos, 2004; Stone, 2014; Wang et al., 2006; Wang & Pickart, 2005; Yee & Goring, 2009): HECT (Homologous to E6-AP C-Terminal), RING (Really Interesting New Gene), and U-box.

The HECT-type ubiquitin ligase is an important class of E3s defined by the presence of a C-terminal catalytic HECT domain. The general features of HECT domains are an N-terminal lobe that contains the E2-binding site and a smaller C-terminal lobe that includes the active-site Cys residue that receives ubiquitin from E2 and links itself with the ubiquitin molecule (Downes et al., 2003; Huibregtse et al., 1995). Classification of HECT E3 proteins into different subfamilies is based on the N-terminal domains (Downes et al., 2003; Grau-Bove, Sebe-Pedros & Ruiz-Trillo, 2013; Marin, 2010; Marin, 2013) responsible for recognizing and binding protein substrates (Kamadurai et al., 2013; Kim et al., 2011; Maspero et al., 2011; Maspero et al., 2013; Rotin & Kumar, 2009), while the conserved C-terminal HECT domain catalyzes the transfer of ubiquitin to various substrates. Substrate proteins usually possess recognition motifs that can directly bind to the N-terminal domains, while the special HECT domains are essential to the prediction and evolution of the *HECT* genes in plants; however, comprehensive research on these genes is limited.

The HECT-type E3 ubiquitin ligases comprise a small class of E3s, and seven genes (*UPL1*-*UPL7*) have been identified in *Arabidopsis thaliana* (Downes et al., 2003). *UPL3* is involved in trichome development (Downes et al., 2003; Patra, Pattanaik & Yuan, 2013), genome endoreduplication (El Refy et al., 2003) and seed size (Miller et al., 2019). *UPL5* is involved in leaf senescence (Miao & Zentgraf, 2010), and *UPL1*, *UPL3*, and *UPL5* in plant immunity (Furniss et al., 2018). These seven *A. thaliana HECT* genes can be classified into five subfamilies or six groups according to the phylogenetic relationships provided in previous studies (Marin, 2013; Meng et al., 2015). However, little research has been conducted on the *HECT* genes in wheat, which is one of the most important crops produced worldwide (Choulet et al., 2014; International Wheat Genome Sequencing Consortium, 2014; International Wheat Genome Sequencing Consortium et al., 2018). In this research, we conducted a comprehensive genome-wide analysis of the wheat *HECT* genes to identify *HECT* genes conserved in wheat, rice, and *A. thaliana*. Gene exon-intron structure, conserved motif, domain structure, chromosomal distribution, duplication event, and expression profile were also analyzed in detail. Our research data will provide useful information for further functional investigation of the *HECT* gene family in allohexaploid wheat and their evolution in polyploid plants.

## Materials & Methods

### Sequence retrieval and identification of the HECT gene family in wheat

To identify the *HECT* genes in wheat, the protein sequences of all HECT genes in *A. thaliana* and rice were retrieved from the Phytozome v13 database (Goodstein et al., 2012) with the Ensembl Plants (Howe et al., 2020) as a complementary sequence database. These protein sequences were then used as queries to conducted local BlastP and tBlastN (Camacho et al., 2009) searches using default parameters (*E*-value <10^−5^) against wheat reference sequence database in the Chinese Spring reference IWGSC RefSeq v1.0 (International Wheat Genome Sequencing et al. 2018) from the Ensembl Plants database. The hmmsearch program in the HMMER software (version 3.3) (Potter et al., 2018) was also used to the identification of *HECT* genes with the HMM profile of the HECT domain (PF00632) in the Pfam 32.0 database (El-Gebali et al., 2019), using the default parameters (*E*-value < 10^−5^). Then, the combined candidate *HECT* genes were used as queries to conduct BlastP and tBlastN searches of the wheat genome again to obtain more potential gene candidates with the default parameters (*E*-value < 10^−5^). The obtained protein sequences were further verified using the InterProScan program (Jones et al., 2014) to confirm the presence of the HECT domain. Finally, each *HECT* gene was revised manually for conserved domain architectures using the Pfam (El-Gebali et al., 2019), PROSITE (Sigrist et al., 2013), and SMART (Letunic & Bork, 2018) databases. Proteins without a typical HECT domain or fewer than 300 amino acids were removed from the final sequence dataset.

### Sequence alignment and phylogenetic analysis

Multiple sequence alignments of the wheat HECT protein sequences were performed by using MUSCLE (Edgar, 2004) with its default parameters, and MAFFT (L-INS-i strategy) (Rozewicki et al., 2019). The phylogenetic tree was constructed and visualized using MEGAX software (Kumar et al., 2018) based on the full-length HECT protein sequences through a neighbor-joining algorithm with 1,000 bootstrap repetitions. The maximum likelihood (ML) methods implemented in PhyML3.1 (Guindon et al., 2010) were also used to construct trees of full-length HECT protein sequences with 1,000 bootstrap repetitions.

### Sequence analysis

The structures of *HECT* genes and the number of exons and introns were determined using the Gene Structure Display Server (Hu et al., 2015) by aligning the coding sequences with their corresponding genomic DNA sequences. The conserved motifs encoded by *HECT* genes were identified using MEME (Multiple EM for Motif Elicitation) (Bailey et al., 2015). The conserved domains of the HECT protein sequences within the phylogenetic trees were visualized and annotated using EvolView (Subramanian et al., 2019).

### Chromosomal location and duplication

To map all *HECT* genes to the wheat chromosomes, information of *HECT* gene chromosomal location was obtained from Ensembl Plants. Gene duplication events of the wheat *HECT* genes were inferred based on their location among the three wheat subgenomes (A, B, and D). Firstly, all-in-all BlastP of the wheat genome was performed to analyze sequence similarity among the three subgenomes (A, B, and D). Secondly, MCScanX (Multiple Collinearity Scan toolkit) (Wang et al., 2012) was then used with default parameters to detect possible gene duplication blocks. Finally, Chromosomal location and syntenic relationships were illustrated using Circos-0.67 (Krzywinski et al., 2009). Synonymous (Ks) and nonsynonymous substitution (Ka) rates were calculated with TBtools (Chen et al., 2018), as previously described (Meng et al., 2015). For each gene pair, the approximate divergence time (T, million years ago, Mya) of the duplication events for each paralogous gene pair was estimated using the mean Ks values from T = Ks/2 *λ*, in which the mean synonymous substitution rate (*λ*) for wheat is 6 ×10^−9^(Wolfe, Li & Sharp, 1987; Wolfe, Sharp & Li, 1989).

### Expression analyses

RNA-Seq data (Accession number “choulet_URGI” and “PRJNA497810”) were downloaded from expVIP (Borrill et al., 2019; Borrill, Ramirez-Gonzalez & Uauy, 2016; Choulet et al., 2014; Ramírez-González et al., 2018) (Table S1–S4) and used to explore the expression patterns of *HECT* genes in wheat. These transcript data were obtained from five organs (root, stem, leaf, spike, and grain) at three developmental stages and flag leaves harvested at 3, 7, 10, 13, 15, 17, 19, 21, 23, and 26 dpa (day post-anthesis). The expression data were log2 based TPM (transcripts per million mapped reads) values, and the heatmap of the expression patterns of wheat *HECT* genes was drawn using the R heatmap.2 function.

## Results

### Identification of the HECT gene family in wheat

To identify *HECT* genes in wheat, the HMM HECT domain profile PF00632 (El-Gebali et al., 2019) and the HECT protein sequences from *A. thaliana* (Downes et al., 2003) and rice (Meng et al., 2015) were used to search against the wheat protein sequences in Ensemble Plants (Howe et al., 2020; International Wheat Genome Sequencing et al. 2018), then the potential *HECT* genes were confirmed by InterProScan (Jones et al., 2014) with Pfam (El-Gebali et al., 2019), Prosite (Sigrist et al., 2013) and SMART (Letunic & Bork, 2018) databases that helped to characterize the candidates by the existence of the complete HECT domain. Ultimately, we identified 25 putative *HECT* genes in the latest wheat genome (Table 1).

**Table 1:** Putative *HECT* genes identified in the wheat genome.

Gene symbol	Gene locus	Chromosome	Gene start (bp)	Gene end (bp)	Gene % GC content	length (AA)
*TaHECT01*	*TraesCS1A02G059500*	1A	40892389	40898032	47.13	1335
*TaHECT02*	*TraesCS1A02G106100*	1A	103311704	103326325	42.01	1010
*TaHECT03*	*TraesCS1A02G288600*	1A	485557978	485566303	43.98	1520
*TaHECT04*	*TraesCS1B02G123400*	1B	149601252	149613588	39.18	1027
*TaHECT05*	*TraesCS1B02G298000*	1B	518258672	518266604	42.38	1521
*TaHECT06*	*TraesCS1D02G108900*	1D	101929905	101943199	39.16	1030
*TaHECT07*	*TraesCS1D02G287600*	1D	386119575	386127952	44.18	1522
*TaHECT08*	*TraesCS2A02G064700*	2A	29095129	29105808	42.87	1862
*TaHECT09*	*TraesCS2B02G076900*	2B	42143087	42153426	43.11	1835
*TaHECT10*	*TraesCS3B02G194900*	3B	215607542	215618805	42.3	695
*TaHECT11*	*TraesCS4A02G285800*	4A	591699693	591709939	41.24	1036
*TaHECT12*	*TraesCS4B02G027200*	4B	20589014	20599094	41.07	1170
*TaHECT13*	*TraesCS4D02G025000*	4D	10931453	10942048	41.32	1122
*TaHECT14*	*TraesCS5A02G121600*	5A	263498258	263513761	42.56	3632
*TaHECT15*	*TraesCS5A02G262600*	5A	475656292	475662357	45.58	832
*TaHECT16*	*TraesCS5B02G112800*	5B	177183624	177199346	42.35	3628
*TaHECT17*	*TraesCS5B02G261000*	5B	445453118	445459346	44.89	858
*TaHECT18*	*TraesCS5D02G118000*	5D	154524415	154539554	42.35	3631
*TaHECT19*	*TraesCS5D02G270200*	5D	373832243	373838247	44.01	817
*TaHECT20*	*TraesCS6A02G003300*	6A	1494577	1504714	46.25	1839
*TaHECT21*	*TraesCS6B02G000300*	6B	163680	173488	45.87	1851
*TaHECT22*	*TraesCS6D02G005600*	6D	2399592	2409297	45.55	1841
*TaHECT23*	*TraesCS7A02G244000*	7A	220125320	220130919	46.62	862
*TaHECT24*	*TraesCS7B02G313300*	7B	559527374	559559604	45.86	897
*TaHECT25*	*TraesCS7B02G496200*	7B	747214104	747221564	42.45	796

### Phylogenetic analysis of HECT genes in wheat

**Figure 1:**
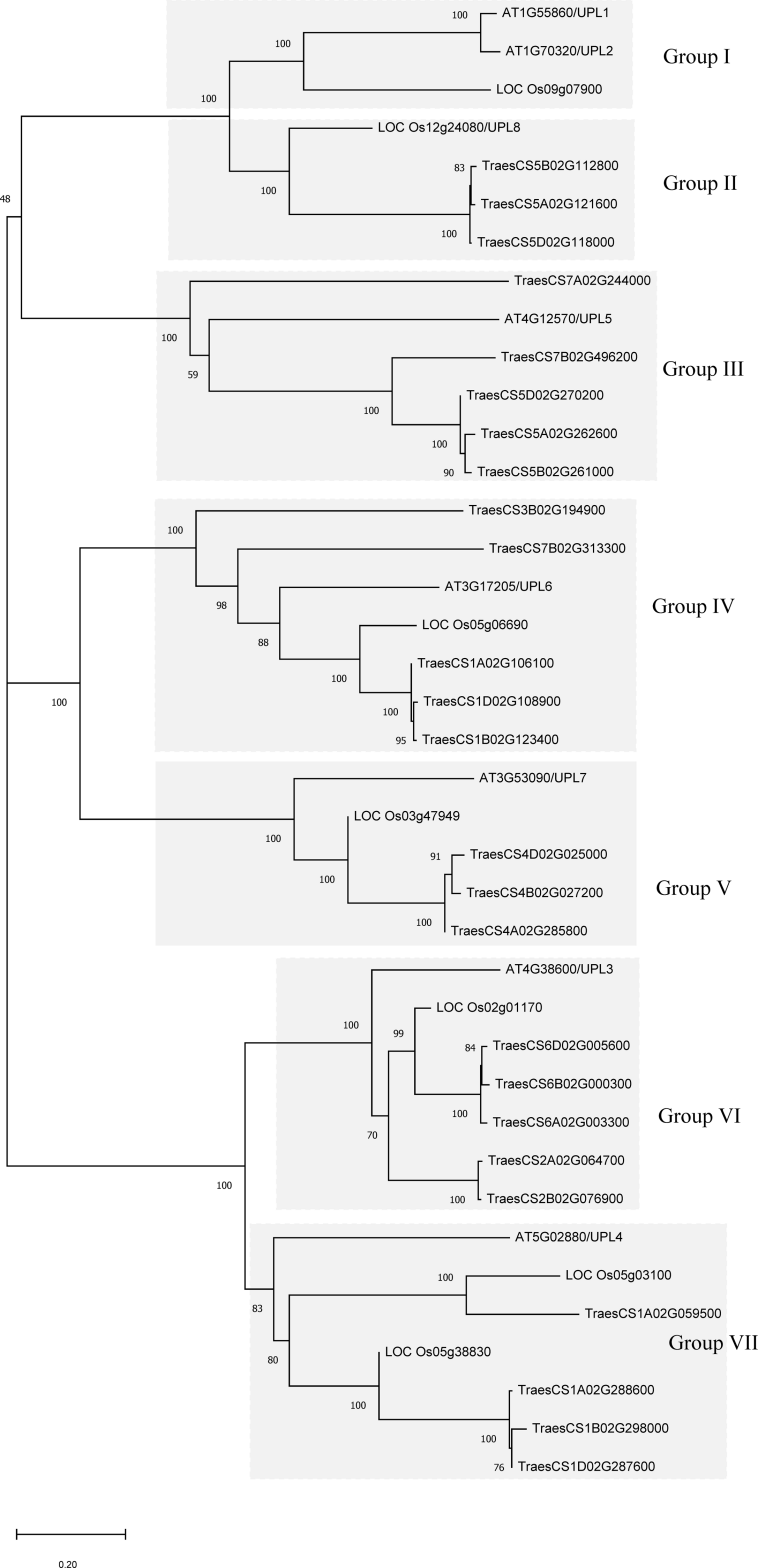
Phylogenetic relationships of 39 *HECT* genes from wheat (25)*,* rice (seven)*,* and *Arabidopsis thaliana* (seven). A neighbor-joining (NJ) unrooted tree is shown and the shaded areas indicate the main branches that correspond to the seven gene groups. MEGAX package was used to construct the NJ tree from full-length amino acid sequence alignments (File S1) of the three plant species, with 1000 bootstrap replicates. Numbers refer to bootstrap support in terms of percentage.

To understand the evolutionary relationship of the wheat *HECT* genes, phylogenetic trees were constructed based on the alignment of the full-length protein sequences and HECT domain sequences of 25 wheat, 7 rice, and 7 *A. thaliana* HECT proteins (Fig. 1 and Fig. S1). According to the classification criteria used for *A. thaliana* and rice in previous studies (Downes et al., 2003; Grau-Bove, Sebe-Pedros & Ruiz-Trillo, 2013; Marin, 2013; Meng et al., 2015), the wheat *HECT* genes were categorized into seven groups (Group I, II, III, IV, V, VI and VII), which contained 0, 3, 5, 5, 3, 5 and 4 *HECT* genes, respectively. Genes in Group III, IV and VI were the most abundant and comprised 60% of the identified genes, while genes in Group I was absent in wheat. Nevertheless, in *A. thaliana*, Group I included two HECT genes, Group II did not include any *HECT* genes, and other Groups included one HECT gene, respectively. These seven groups can be further classified into five subfamilies that correspond to those described in a previous study (Marin, 2013).

### Gene exon-intron structure and conserved motif and domain architecture of the wheat HECT genes

**Figure 2:**
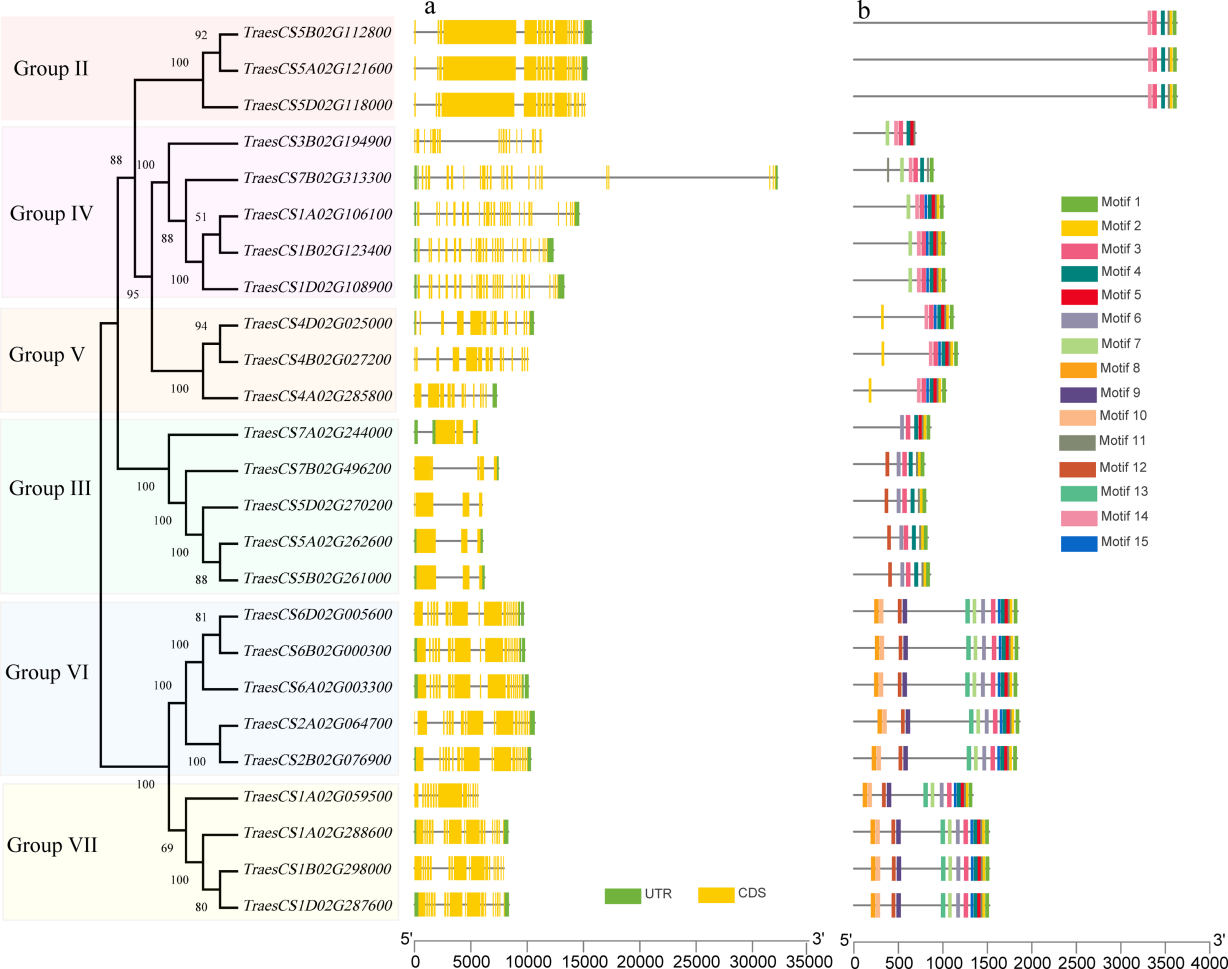
Gene structures and conserved motifs that encode 25 wheat HECT proteins based on phylogenetic relationships. The unrooted neighbor-joining tree was constructed using the alignment of full-length amino acid sequences (File S2) with the MEGAX package. The lengths of the exons and introns in each *HECT* gene are displayed proportionally. The green boxes, yellow boxes, and lines indicate UTRs, exons, and introns, respectively. Conserved motifs are showed in different colors.

**Figure 3:**
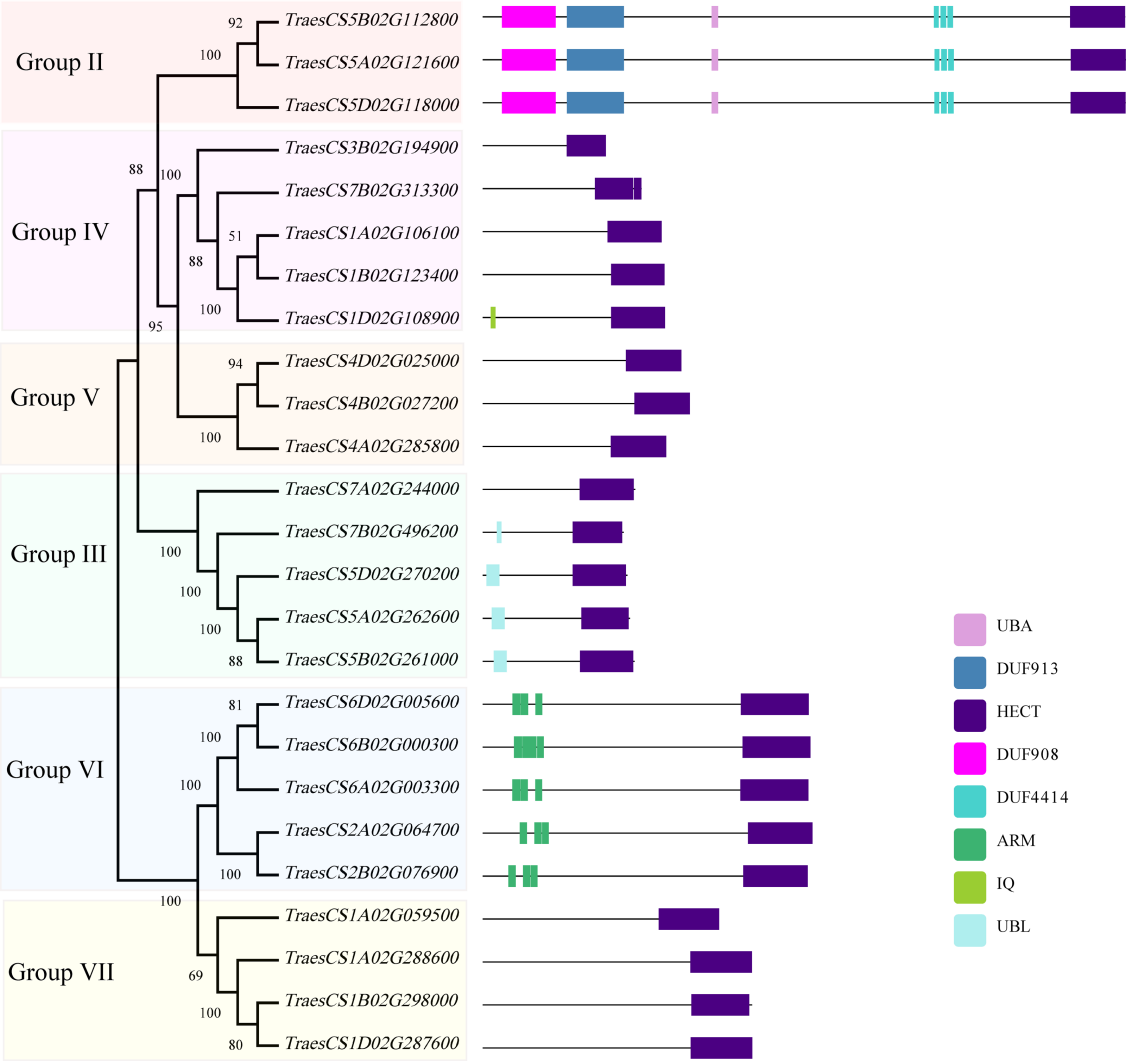
Domain architecture of 25 wheat HECT proteins according to their phylogenetic relationships. Each domain is represented by a colored box. UBA: Ubiquitin associated domain, DUF: Domain of Unknown Function, ARM: Armadillo repeats, IQ; IQ Short calmodulin-binding motif, UBL: Ubiquitin-like domain.

To investigate the structural characteristics of wheat *HECT* genes, the exon-intron structures of the wheat *HECT* genomic sequences, conserved motifs, and the domain architecture of the wheat HECT proteins were compared based on their phylogenetic relationships. Each gene structure was revealed by aligning its coding sequences with the corresponding genomic sequences (Chen et al., 2018; Hu et al., 2015). Most of the wheat *HECT* genes contained abundant (more than ten) exons and only those in Group III had only three or four exons (Fig. 2A). Closely related *HECT* genes in the same phylogenetic group had similar exon-intron structures, and those with closer evolutionary relationships were more similar in their number and length of exons and introns. The conserved motifs of wheat HECT proteins in each group were analyzed using MEME software (Bailey et al., 2015). Fifteen conserved motifs (motif1-motif15) were predicted and these motifs were specific to each group (Fig. 2B). The composition of the structural motifs varied among the different HECT groups, while similar motifs were found in the same group. Additionally, the motifs encoding the HECT domain in the C-terminal regions of wheat HECT proteins were relatively conserved, suggesting that the functions of the HECT proteins were intergroup specific. The domain architecture of HECT proteins was obtained using the InterProScan program (Jones et al., 2014) with a three-database annotation (El-Gebali et al., 2019; Letunic & Bork, 2018; Sigrist et al., 2013). In addition to the HECT domain, other domains were found in the N-terminal regions of wheat HECT proteins (Fig. 3). The wheat *HECT* genes that were derived from the same group generally had similar exon-intron structures (Fig. 2A), motif compositions (Fig. 2B), and domain architectures (Fig. 3).

### Chromosomal location and duplication of wheat HECT genes

**Figure 4:**
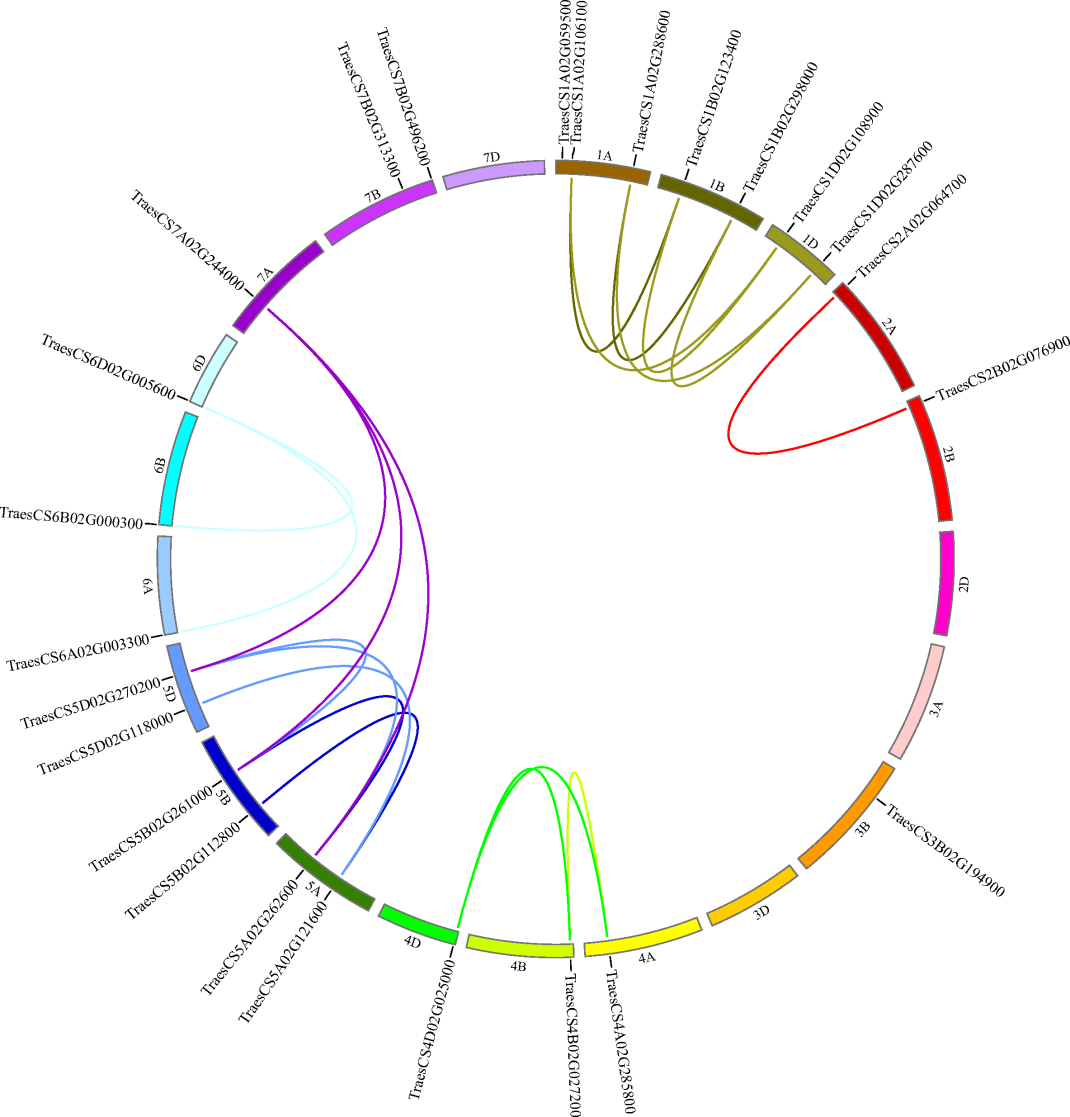
Chromosome locations of *HECT* genes and segmentally duplicated gene pairs in the wheat genome. Chromosomes are shown in different colors and in a circular form. The approximate distribution of each wheat *HECT* gene is marked on the circle with a short black line. Colored curves denote the details of syntenic regions between the wheat *HECT* genes. The purple curves represent the estimated time of duplication events that occurred 100–112 Mya, and the other curves represent the estimated time of duplication events 2–12 Mya.

To decide the chromosomal locations of the wheat *HECT* genes, the 25 putative wheat *HECT* genes were located in the 21 chromosomes of the wheat genome database available from Ensembl Plants (Howe et al., 2020; International Wheat Genome Sequencing et al. 2018). The wheat *HECT* genes were randomly distributed in 17 of 21 chromosomes; chromosome 2D, 3A, 3D, and 7D contained no *HECT* genes, chromosome 1A contained three *HECT* genes, chromosome 1B, 1D, 5A, 5B, 5D, and 7B each contained two *HECT* genes, and the other chromosomes each contained only one *HECT* gene (Fig. 4). The 25 wheat *HECT* genes were approximately evenly distributed among the A (9), B (10), and D (6) subgenomes, which was in accordance with the observation that most *HECT* genes have three homoeologous sequences located across three subgenomes. However, the *HECT* genes were not randomly distributed among the different chromosomal groups of the three subgenomes. The chromosomal Group II, III, and VII contained two, one, and three sequences, respectively. The remaining 19 sequences were more evenly distributed across chromosomal Group I, IV, V, and VI, and ranged from three to seven genes per group. An interesting finding was that the location of the HECT genes on chromosome 4A was opposite with those of the homoeologous genes on chromosome 4B and chromosome 4D (Fig. 4).

Segmental and tandem duplication are considered two essential factors for gene family expansion in plants (Cannon et al., 2004; Panchy, Lehti-Shiu & Shiu, 2016; Qiao et al., 2019; Zhu et al., 2014). To examine duplication patterns of the wheat *HECT* genes, we identified tandem and segmental duplication events using MCscanX (Wang et al., 2012) employing default parameters with TBtools (Chen et al., 2018). No tandem duplicated HECT gene pairs were identified in the 25 wheat *HECT* genes; however, 21 of the 25 wheat *HECT* genes were involved in segmental duplication. Twenty segmental duplicated HECT gene pairs were identified (Fig. 4 and Table 2), indicating that the segmental duplication events had contributed to *HECT* gene family expansion. To date the gene duplication time of these segmentally duplicated *HECT* genes, the Ks and Ka distances, as well as the Ka/Ks ratios were calculated. The Ka/Ks ratios for segmentally duplicated HECT gene pairs ranged from 0.07 to 0.44, with an average value of 0.20 (Table 2), implying that these segmentally duplicated *HECT* genes were under purifying selection, as indicated by the Ka/Ks ratios were less than 1. The divergence time of duplication events were inferred by Ks (Table 2). Results showed that within six existed phylogenetic groups, the two closest wheat *HECT* genes were duplicated about 2–12 million years ago (Mya), while the other genes were duplicated about 100–112 Mya.

**Table 2:** Estimates of the segmental duplication events in the wheat *HECT* gene pairs.

Group	Gene 1	Gene 2	Ka	Ks	Ka/Ks	Mya
II	*TraesCS5A02G121600*	*TraesCS5B02G112800*	0.01	0.04	0.16	2.93
	*TraesCS5A02G121600*	*TraesCS5D02G118000*	0.00	0.03	0.14	2.87
III	*TraesCS5A02G262600*	*TraesCS5B02G261000*	0.01	0.08	0.18	6.29
	*TraesCS5A02G262600*	*TraesCS5D02G270200*	0.01	0.07	0.18	6.09
	*TraesCS5A02G262600*	*TraesCS7A02G244000*	0.55	1.34	0.41	111.99
	*TraesCS5B02G261000*	*TraesCS5D02G270200*	0.01	0.06	0.23	4.79
	*TraesCS5B02G261000*	*TraesCS7A02G244000*	0.55	1.29	0.42	107.74
	*TraesCS5D02G270200*	*TraesCS7A02G244000*	0.55	1.24	0.44	103.15
IV	*TraesCS1A02G106100*	*TraesCS1B02G123400*	0.00	0.04	0.07	3.07
	*TraesCS1A02G106100*	*TraesCS1D02G108900*	0.00	0.03	0.15	2.44
	*TraesCS1B02G123400*	*TraesCS1D02G108900*	0.00	0.03	0.17	2.29
V	*TraesCS4A02G285800*	*TraesCS4B02G027200*	0.01	0.03	0.19	2.91
	*TraesCS4A02G285800*	*TraesCS4D02G025000*	0.02	0.05	0.30	4.37
	*TraesCS4B02G027200*	*TraesCS4D02G025000*	0.01	0.06	0.24	5.04
VI	*TraesCS2A02G064700*	*TraesCS2B02G076900*	0.00	0.03	0.15	2.66
	*TraesCS6A02G003300*	*TraesCS6D02G005600*	0.01	0.12	0.08	10.01
	*TraesCS6B02G000300*	*TraesCS6D02G005600*	0.01	0.13	0.07	11.03
VII	*TraesCS1A02G288600*	*TraesCS1B02G298000*	0.01	0.06	0.23	5.41
	*TraesCS1A02G288600*	*TraesCS1D02G287600*	0.01	0.05	0.11	4.25
	*TraesCS1B02G298000*	*TraesCS1D02G287600*	0.01	0.08	0.17	6.43

**Notes.**

Ks synonymous substitution rate Ka nonsynonymous substitution rate Mya million years ago

### Expression profiles of wheat HECT genes

**Figure 5:**
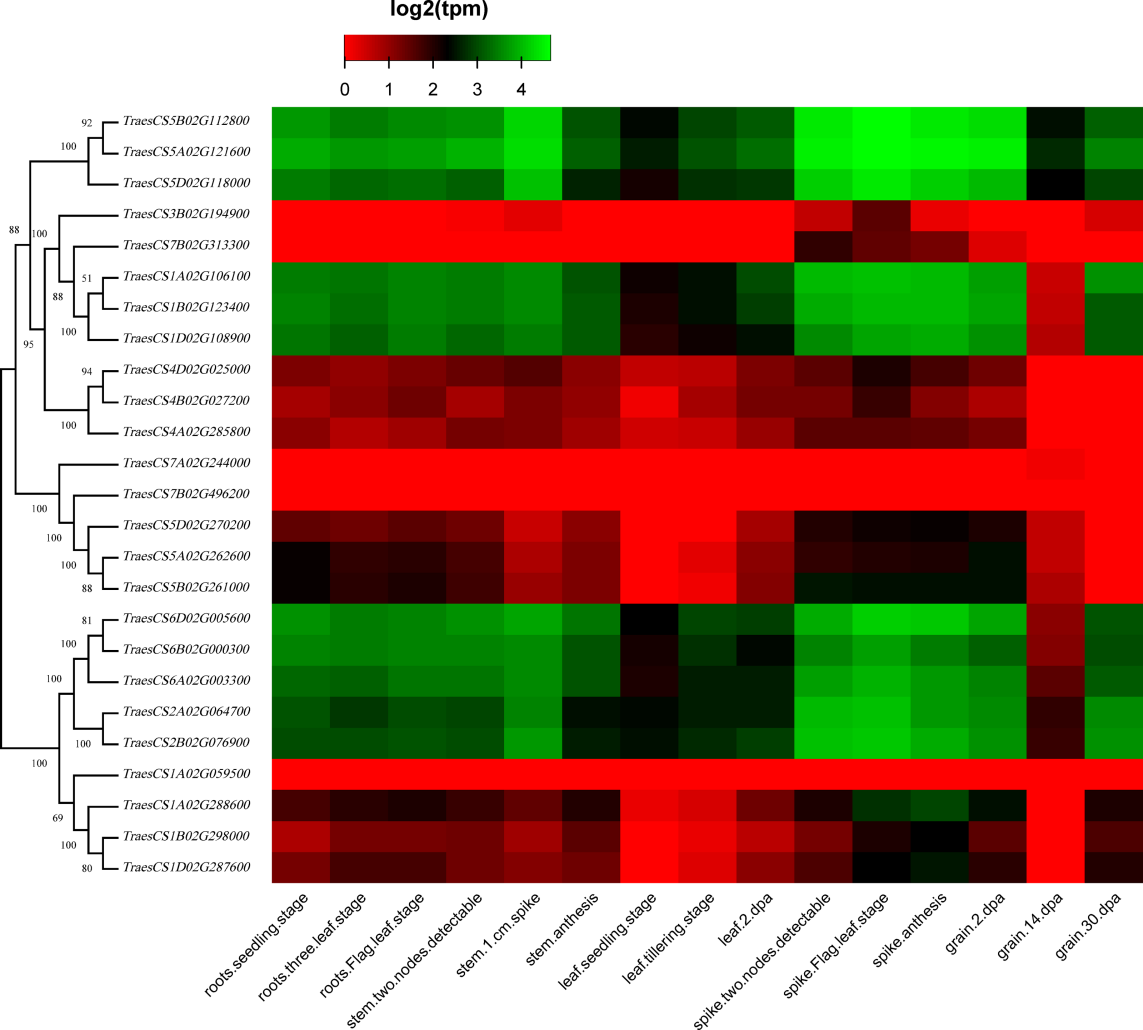
Heatmap of wheat *HECT* gene expression patterns in 15 different tissues. Transcriptional levels were obtained from expVIP. The RNA-seq relative expression data from 15 tissues was used to reconstruct the expression patterns of the wheat genes. The sources of the samples are provided on the *x*-axis.

**Figure 6:**
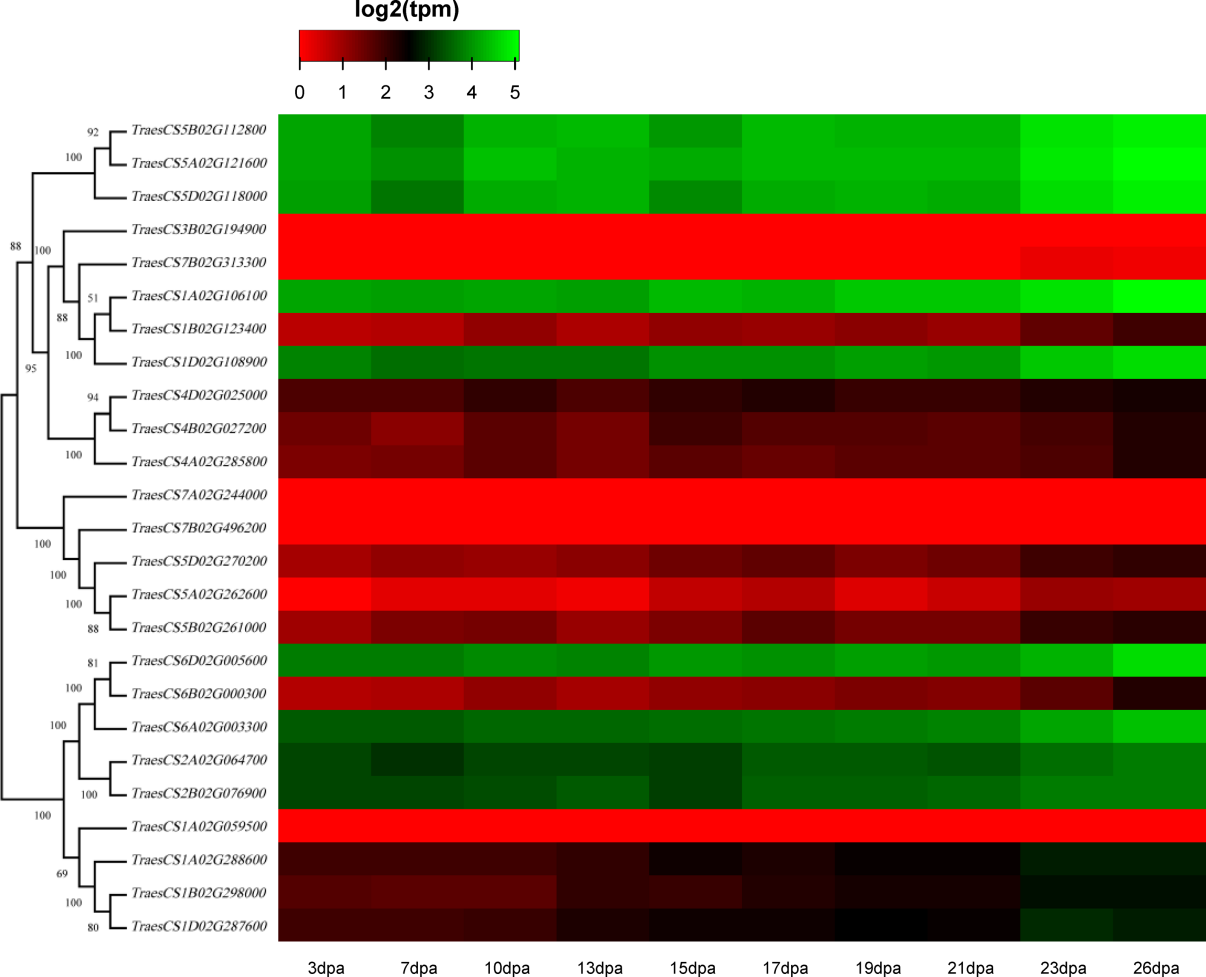
Heatmap of wheat HECT gene expression patterns in wheat leaf senescence. Transcriptional levels were obtained from expVIP. The RNA-seq relative expression data from flag leaves harvested at 3, 7, 10, 13, 15, 17, 19, 21, 23, and 26 dpa (day post-anthesis) was used to reconstruct the expression patterns of the wheat genes. The sources of the samples are provided on the *x*-axis.

To discover the potential roles of these wheat *HECT* genes in growth and development, we used public RNA-seq data covering 15 tissues at different growth stages from expVIP (Borrill et al., 2019; Borrill, Ramirez-Gonzalez & Uauy, 2016; Ramírez-González et al., 2018). Based on the wheat RNA-seq data, the 25 wheat *HECT* genes were detected in all 15 tissues at the gene level (Fig. 5, Table S1, and Table S2). Moreover, the expression of these genes exhibits distinct expression and tissue-specific characteristics. Most *HECT* genes in Group II, IV, and VI were relatively highly expressed in the roots, stems and spikes, while those in the leaves were expressed at relatively low levels (Fig. 5). Interestingly, in wheat grain tissues, most wheat *HECT* gene expression in Group II, IV, and VI were high at 2 dpa and 30 dpa and low at 14 dpa. Moreover, genes within each group or in different groups had similar expression patterns in different tissues, such as the high expression of genes in Group II (*TraesCS5A02G121600*, *TraesCS5B02G112800*, *TraesCS5D02G118000*), Group IV (*TraesCS1A02G106100*, *TraesCS1B02G123400*, *TraesCS1D02G108900*), and Group VI (*TraesCS6A02G003300*, *TraesCS6B02G000300*, *TraesCS6D02G005600*, *TraesCS2A02G064700*, and *TraesCS2B02G076900*), except for *TraesCS3B02G194900* and *TraesCS7B02G313300* (Fig. 5). Furthermore, the genes in Group II, IV, and VI were relatively highly expressed in the spikes at different developmental stages and in stems at the one cm spike stage compared to those in other tissues (Fig. 5). According to RNA-seq data of the ten-time point expression time course of wheat senescence in the flag leaf, the expression level of most wheat HECT genes in Group II, IV, and VI gradually increased with the increase of dpa (Fig. 6, Table S3, and Table S4).

## Discussion

*HECT* genes play important roles in *A. thaliana* and diverse plant growth, developmental and physiological processes (Downes et al., 2003; (El Refy et al., 2003); Furniss et al., 2018; Miao & Zentgraf, 2010; Miller et al., 2019; Patra, Pattanaik & Yuan, 2013), including trichome development (Downes et al., 2003), genome endoreduplication (El Refy et al., 2003), seed size (Miller et al., 2019), leaf senescence (Miao & Zentgraf, 2010), and plant immunity (Furniss et al., 2018). However, this gene family has not been investigated in wheat. In this research, we conducted an extensive analysis of the wheat *HECT* genes, including their evolution, gene exon-intron structure, conserved motif, domain structure, chromosomal location, duplication event, and expression pattern.

We identified 25 *HECT* genes in the wheat genome, which is 3.6 times the number present in *A. thaliana* (Downes et al., 2003). However, a former study discovered 19 soybean *HECT* genes, which is 2.7 times the number found in *A. thaliana* (Meng et al., 2015). Our results showed six more *HECT* genes in wheat than the number previously estimated in the soybean genome. A possible explanation for this difference is that wheat is a hexaploid crop with 21 chromosomes containing three subgenomes (A, B, and D) (International Wheat Genome Sequencing Consortium, 2014; International Wheat Genome Sequencing Consortium et al., 2018), while soybean is a diploid crop with 20 chromosomes derived from an ancient tetraploid that may have had about two times more the number of *HECT* genes than other normal diploid species (Schmutz et al., 2010).

The phylogenetic analysis of the 25 wheat *HECT* genes classified them into subfamilies similar to those characterized by previous research (Downes et al., 2003; Grau-Bove, Sebe-Pedros & Ruiz-Trillo, 2013; Marin, 2013; Meng et al., 2015). The classification was according to the corresponding HECT gene sequence homology. Based on the phylogenetic relationships among the *HECT* genes in wheat, rice, and *A. thaliana*, the wheat *HECT* genes were classified into seven groups. Compared with a former report in *A. thaliana* (Marin, 2013), subfamily IV *HECT* genes were absent in wheat. Wheat subfamily V (Group I, *UPL1/2* and Group II, *UPL8* in this study) contained three genes, subfamily VI (Group III, *UPL5*) contained five genes, subfamily III (Group IV, *UPL6*) contained five genes, Subfamily II (Group V, *UPL7*) contained three genes, and subfamily I (Group VI, *UPL3* and Group VII, *UPL4*) contained nine genes. The *HECT* gene Group I was not observed in the wheat genome. With the exception of Group I and II, other wheat Groups own HECT genes orthologous with *A. thaliana*. This is basically consistent with the results of a previous HECT gene investigation in plants (Marin, 2013), suggesting that *A. thaliana* HECT gene Group II (*UPL8* in this study) was lost, while the wheat HECT gene Group I (*UPL1/UPL2*) was not observed in our analysis. Gene members of each phylogenetic Group often possess identical gene exon-intron structures, conserved motifs, and domain architectures, indicating that they probably recognize, bind, and might interact with same or similar substrate protein.

Segmental duplication events, tandem duplication events, as well as transposition events are three main evolutionary mechanisms of duplication events that expand the members of gene family (Cannon et al., 2004; Panchy, Lehti-Shiu & Shiu, 2016; Qiao et al., 2019; Zhu et al., 2014). Segmental duplications frequently occur in higher plants, because they are diploidized polyploids that have maintained various duplicated chromosomal blocks in the existing genomes (Cannon et al., 2004; Qiao et al., 2019). In this present research, we discovered that 21 of the 25 wheat *HECT* genes were located in chromosomes across the three subgenomes (A, B, D), indicating that segmental duplication obviously contributed to expanding the wheat *HECT* gene family. A previous study has shown that the allohexaploid wheat subgenomes A, B, and D were originally derived from three diploid (2*x*; 2*n* = 14) species and underwent three hybridization events (International Wheat Genome Sequencing 2014). The A and B subgenomes diverged from a common ancestor ∼7 million years ago and the first hybridization occurred ∼5.5 million years ago between A and B subgenomes, leading to the D subgenome through homoploid hybrid speciation. The second hybridization between the A and B subgenomes gave rise to the AABB genome <0.8 million years ago via polyploidization. Wheat originated <0.4 million years ago by allopolyploidization from a third hybridization. By estimating the approximate dates of the segmentally duplicated pairs of wheat *HECT* genes, we infer that the paralogous genes in wheat HECT groups originated from a relatively recent duplication event during the shaping of the three subgenomes (A, B, D) that occurred before the second hybridization event in wheat evolution history, except for *TraesCS7A02G244000* in Group III, which originated from a relatively ancient duplication event before the appearance of the common ancestor of the A and B subgenomes. Thus, segmental duplication events were the primary driving forces for *HECT* gene evolution during the speciation and evolution of allohexaploid wheat.

To better understand the roles of the *HECT* genes during the life cycle of wheat, we performed an expression analysis of public RNA-seq data (Borrill, Ramirez-Gonzalez & Uauy, 2016; Choulet et al., 2014; Ramírez-González et al., 2018) in 15 tissues at different developmental stages. Analysis of the expression patterns of these wheat genes in 15 tissues showed that most wheat *HECT* genes in Group II, IV, and VI were relatively highly expressed in the roots, stems, and spikes. In particular, the genes in Groups II, IV, and VI were relatively highly expressed in the spikes at different developmental stages and in stems at the 1-cm spike stage compared to other tissues. Therefore, the expression of these genes may be closely related to wheat spike growth and development, suggesting that the *HECT* genes in highly expressed spikes may be involved in the regulation or degradation of proteins via ubiquitination during spike development stage. Previous studies have revealed that *A. thaliana AT4G38600/UPL3* plays a specific role during trichome development (Downes et al., 2003; Patra, Pattanaik & Yuan, 2013) and seed size (Miller et al., 2019) and that *AT4G12570*/*UPL5* is an important transcription factor that positively regulates leaf senescence by the ubiquitination and degradation of *AT4G23810*/*WRKY5 3* (Miao & Zentgraf, 2010). In our investigation, the wheat genes orthologous to *A. thaliana AT4G38600*/*UPL3* included five paralogous genes in Group VI. Except for grain at the 14 dpa stage, these five genes were all relatively highly expressed in wheat, particularly in spikes. A reasonable explanation is that the relatively low expression of *UPL3* at 14 dpa stage may be related to the size of wheat seeds and is an adaptive regulation mechanism during seed formation. This is consistent with a recent study of *UPL3* in *Brassica napus* (Miller et al., 2019). Miller et al. determined a mechanism in which the proteasomal degradation of LEC2, a transcription factor controlling seed maturation, is mediated by UPL3 and reduced *UPL3* expression would increase LEC2 protein levels and seed size. The wheat genes orthologous to *A. thaliana AT4G12570*/*UPL5* were five paralogous genes in Group III, which were expressed in different wheat tissues but showed distinct features. At different developmental stages, the expression levels of *TraesCS5D02G270200*, *TraesCS5A02G262600*, and *TraesCS5B02G261000* in roots, stems, and spikes were relatively unchanged, but gradually increased in leaves and decreased in grain. The three wheat genes *TraesCS1A02G106100*, *TraesCS1B02G123400*, and *TraesCS1D02G108900* are orthologous to *A. thaliana AT3G17205/UPL6* in Group IV, and the genes in Group II (*UPL8* absent in *A. thaliana*) showed similar expression patterns to those in Group VI (*UPL3*). RNA-seq data of wheat leaf senescence (Borrill et al., 2019) indicated that HECT genes in Group II, IV and VI (*UPL8*, *UPL6*, and *UPL3*) might also play crucial roles in plant leaf senescence. The differential expression of paralogous HECT genes in or among groups in wheat suggests that they might have the same or similar functions as their orthologous genes in *A. thaliana* and *Brassica napus*; nevertheless, they might have evolved functional differences.

A former research discovered that AT4G38600/UPL3 mediated UPS-dependent proteolysis of the two transcription factors AT5G41315/GL3 and AT1G63650/EGL3*,* which interact with the ARM domains of UPL3 and function as positive regulators during *A. thaliana* trichome development (Patra, Pattanaik & Yuan, 2013). The evolutionarily closely related Group VII (UPL4) and VI (UPL3) belong to the same subfamily I defined in previous studies (Grau-Bove, Sebe-Pedros & Ruiz-Trillo, 2013; Marin, 2013) and genes in Group VII contain no ARM domains (Fig. 3) and thus, are differentially expressed at relatively low levels (Fig. 5) compared to those genes in Group VI. More functional explorations of these genes could improve our understanding of the roles of *HECT* genes in wheat and other plants during growth and development.

## Conclusions

Herein, 25 identified wheat HECT genes were classified into six phylogenetic groups and distributed evenly in 17 of 21 chromosomes of the three subgenomes. Twenty-one hypothesized segmentally duplicated genes indicated that segmental duplication was significantly associated with the expansion of these HECT genes. The expression analysis revealed that most wheat HECT genes in Group II, IV, and VI (*UPL8*, *UPL6*, and *UPL3*) were highly expressed in roots, stems, and spikes at different developmental stages and gradually increased with the increase of dpa, suggesting that these genes may be involved in wheat growth, development and leaf senescence. This study provides useful information for further biological functional analysis of the HECT gene family in allohexaploid wheat.

##  Supplemental Information

10.7717/peerj.10457/supp-1 Supplemental Information 1 Phylogenetic relationships of 39 HECT genes from wheat (25), rice (7), and Arabidopsis thaliana (7) A neighbor-joining (NJ) unrooted tree is shown and the shaded areas indicate the main branches that correspond to the seven gene groups. MEGAX package was used to construct the NJ tree from domain sequence alignments (File S4) of the three plant species, with 1000 bootstrap replicates. Numbers refer to bootstrap support in terms of percentage.****

10.7717/peerj.10457/supp-2 Supplemental Information 2 TPM transcription count data for 25 wheat HECT genes in 15 tissues

10.7717/peerj.10457/supp-3 Supplemental Information 3 TPM log2-transfromed transcription count data for 25 wheat HECT genes in 15 tissues

10.7717/peerj.10457/supp-4 Supplemental Information 4 TPM transcription count data for 25 HECT genes in wheat leaf senescence

10.7717/peerj.10457/supp-5 Supplemental Information 5 TPM log2-transfromed transcription count data for 25 HECT genes in wheat leaf senescence

10.7717/peerj.10457/supp-6 Supplemental Information 6 FASTA format multiple sequence alignments of the 39 full-length HECT proteins in wheat (25)*,* rice (7)*,* and *Arabidopsis thaliana* (7)

10.7717/peerj.10457/supp-7 Supplemental Information 7 FASTA format multiple sequence alignments of 25 full-length HECT proteins in wheat

10.7717/peerj.10457/supp-8 Supplemental Information 8 Classification information of 25 HECT genes in wheat

10.7717/peerj.10457/supp-9 Supplemental Information 9 HECT domain sequence alignments of 39 HECT genes from wheat (25), rice (7), and Arabidopsis thaliana (7)

## Additional Information and Declarations

### Data Availability

The following information was supplied regarding data availability: The raw data is available in the Supplemental Files.
