# Supplementary figures and images for: Genome-wide identification and evolution of HECT genes in wheat
Source: PeerJ. 2020 Dec 2;8:e10457. doi: 10.7717/peerj.10457 (PMC7718792; doi:10.7717/peerj.10457)

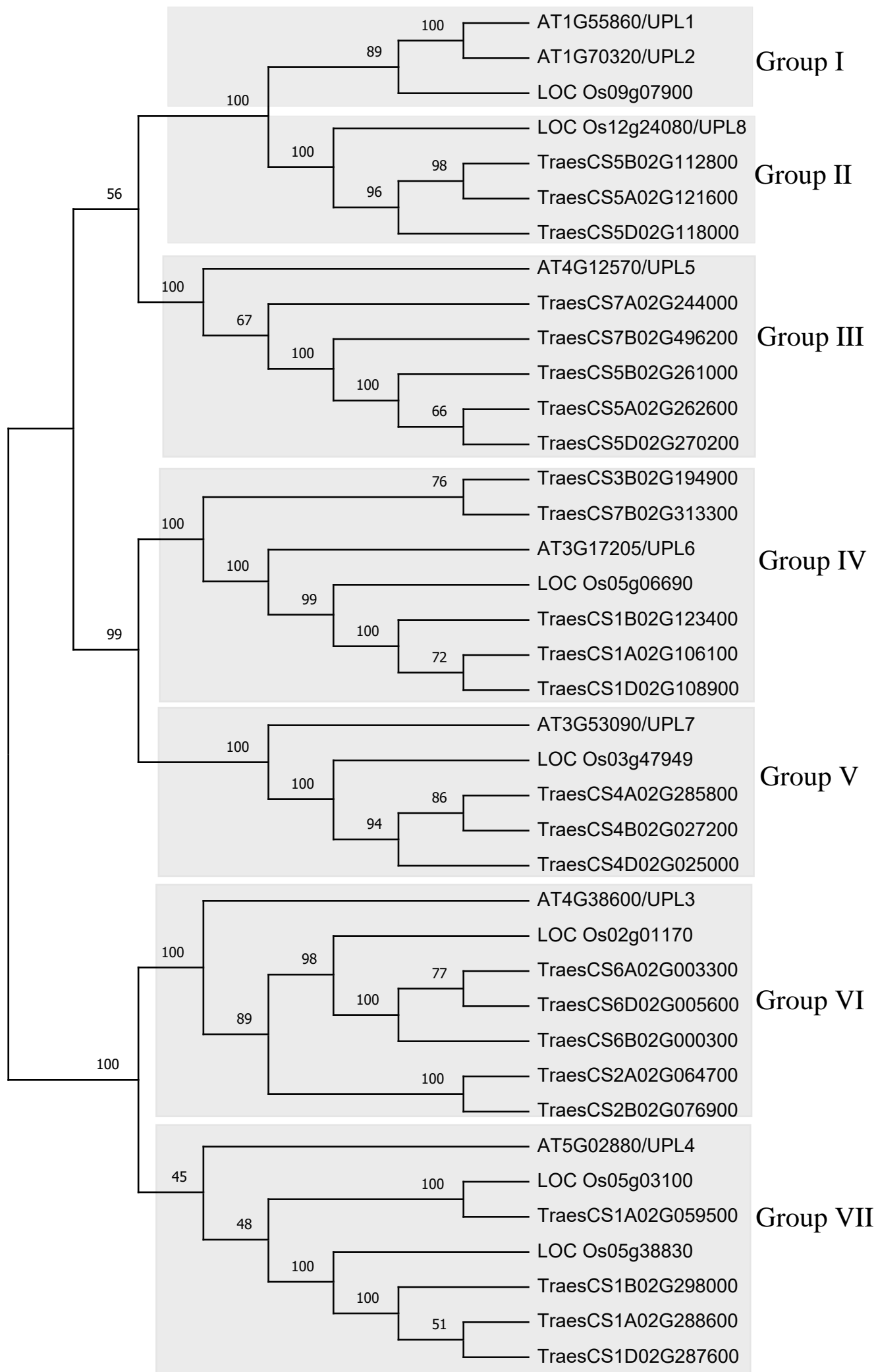

Supplement: Supplemental Information 1 — A neighbor-joining (NJ) unrooted tree is shown and the shaded areas indicate the main branches that correspond to the seven gene groups. MEGAX package was used to construct the NJ tree from domain sequence alignments (File S4) of the three plant species, with 1000 bootstrap replicates. Numbers refer to bootstrap support in terms of percentage. [file peerj-08-10457-s001.pdf]
